# Homologous point transformer for multi-modality prostate image registration

**DOI:** 10.7717/peerj-cs.1155

**Published:** 2022-12-01

**Authors:** Alexander Ruchti, Alexander Neuwirth, Allison K. Lowman, Savannah R. Duenweg, Peter S. LaViolette, John D. Bukowy

**Affiliations:** 1 Department of Electrical Engineering and Computer Science, Milwaukee School of Engineering, Milwaukee, WI, United States; 2 Department of Radiology, Medical College of Wisconsin, Milwaukee, WI, United States; 3 Department of Biophysics, Medical College of Wisconsin, Milwaukee, WI, United States; 4 Department of Biomedical Engineering, Medical College of Wisconsin, Milwaukee, WI, United States

**Keywords:** Registration, Transformer, Deep learning, Medical imaging, Control points

## Abstract

Registration is the process of transforming images so they are aligned in the same coordinate space. In the medical field, image registration is often used to align multi-modal or multi-parametric images of the same organ. A uniquely challenging subset of medical image registration is cross-modality registration—the task of aligning images captured with different scanning methodologies. In this study, we present a transformer-based deep learning pipeline for performing cross-modality, radiology-pathology image registration for human prostate samples. While existing solutions for multi-modality prostate image registration focus on the prediction of transform parameters, our pipeline predicts a set of homologous points on the two image modalities. The homologous point registration pipeline achieves better average control point deviation than the current state-of-the-art automatic registration pipeline. It reaches this accuracy without requiring masked MR images which may enable this approach to achieve similar results in other organ systems and for partial tissue samples.

## Introduction

Prostate cancer is the second most common cause of cancer-related deaths among men in the United States (Siegel, Miller & Jemal, 2019). The current paradigm for prostate cancer diagnosis relies on targeted biopsies which are reliable but invasive. Fortunately, in up to 27% of cases, biopsy can be avoided through MRI diagnoses (Slomski, 2017). And magnetic resonance imaging (MRI) is becoming the gold standard for biopsy guidance. Previous studies have shown promise in using deep learning models to assist in the identification of prostate cancer in magnetic resonance (MR) images. These studies have relied on radiologist-annotated MR images to classify clinically significant prostate cancer (Saha, Hosseinzadeh & Huisman, 2021). Other studies have used data sets with pixel-wise labeled cancer regions in histological ground truth to train MRI cancer diagnosis models (McGarry et al., 2019; Seetharaman et al., 2021). While histological ground truth provides higher resolution and enables annotation through the most reliable diagnostic technique, these data sets are limited in both size and breadth due to the cost of labeling the ground truth in the histology domain and manual registration of labels across imaging modalities. Automatic transference of pixel-wise cancer annotation maps from histology into MR space would unlock far more training data for cancer-predictive deep learning models that operate on MR images.

Alignment (registration) between MRI and histology slides must be resolved in order to transfer pathologist-generated cancer annotations into MR space. This image registration task is made difficult by the deformations of the prostate that occur between the MRI scans and the imaging of the histology slides (Shao et al., 2021a). The sources for these deformations include endorectal coil pressure, inconsistencies between the histology slicing jig and the *in vivo* suspension of the prostate, and folding and tearing of the sliced tissue (Hurrell et al., 2017). Because of these deformations, MRI and histology registration algorithms currently require the use of both affine and nonlinear transformations to optimally align the images. Previously, a manual registration process was used to solve for these transformations in the data set. An expert annotator would place corresponding points on MR images and their matching histology slices (Hurrell et al., 2017; McGarry et al., 2018, 2019). Then, transformations were resolved using the corresponding (homologous) points and applied to the histology images (Meyer et al., 2006). Due to human interaction, this technique is both time intensive and prone to inaccuracy.

### Background

Previous attempts to automate the prostate image registration process through the use of iterative computational methods and deep learning have resulted in moderate success (Sood et al., 2021; Shao et al., 2021b). The recent iterative method RAPSODI reconstructs the three-dimensional prostate volume from two-dimensional slices (Rusu et al., 2020). To find reasonable initial alignments for each slide, the slices are registered to their adjacent histology slices. Then, RAPSODI iteratively estimates optimal affine and nonlinear transformations using gradient descent with sum of squared differences and Mattes mutual information as loss functions. While this method is robust, it is computationally expensive and requires careful manual adjustment of several hyperparameters. ProsRegNet, a deep learning-based alternative to RAPSODI, directly estimates parameters for affine and thin plate spline transforms using a neural network (Shao et al., 2021a). The design is largely patterned after CNNGeometric (Rocco, Arandjelovic & Sivic, 2017), a general image registration pipeline, and uses ResNet-101 (He et al., 2016), a pre-trained feature extraction convolutional neural network (CNN), to create feature maps from both histology and MR images. A custom correlation layer is used to capture the relationships between the sectors of these feature maps before a second convolutional neural network directly predicts the parameters of ProsRegNet’s transforms. While ProsRegNet significantly improves upon RAPSODI’s inference time, the resolution of the feature maps it uses is limited due to the correlation layer’s high memory consumption. Furthermore, this network structure is optimized for aligning the masks of the MR and histology images at the cost of intra-prostate alignment. In 2021, Shao et al. (2021b) applied a weakly-supervised deep learning approach to improve on the results of ProsRegNet. The network they developed used two separate convolutional input heads that are concatenated and followed by dense layers to estimate affine and nonlinear transformable parameters. It was trained with both masked and unmasked MR images and beat ProsRegNet in all common registration metrics. Despite this, the network is still fundamentally limited by the precision of a 4 × 4 thin plate spline transform.

### Study goals

In this study, we propose a new cross-modality registration pipeline that relies on a deep learning network to estimate homologous points on histology and MR images. After the network predicts homologous points, the proposed pipeline uses the estimated points to fit a thin plate spline transform and warp the histology into MR space. This pipeline provides three novel advantages over previously described approaches. First, this pipeline is transform-invariant; any image transform that can be resolved from two point clouds may be used in registration. Second, while most automated registrations pipelines require masked MR images, this approach can register completely unmasked data. Finally, the homologous point pipeline allows for more flexibility in registration fine-tuning through point cloud post-processing than the previous approaches while maintaining the inference time advantage of deep learning.

## Methods

### Overview

**Figure 1:**
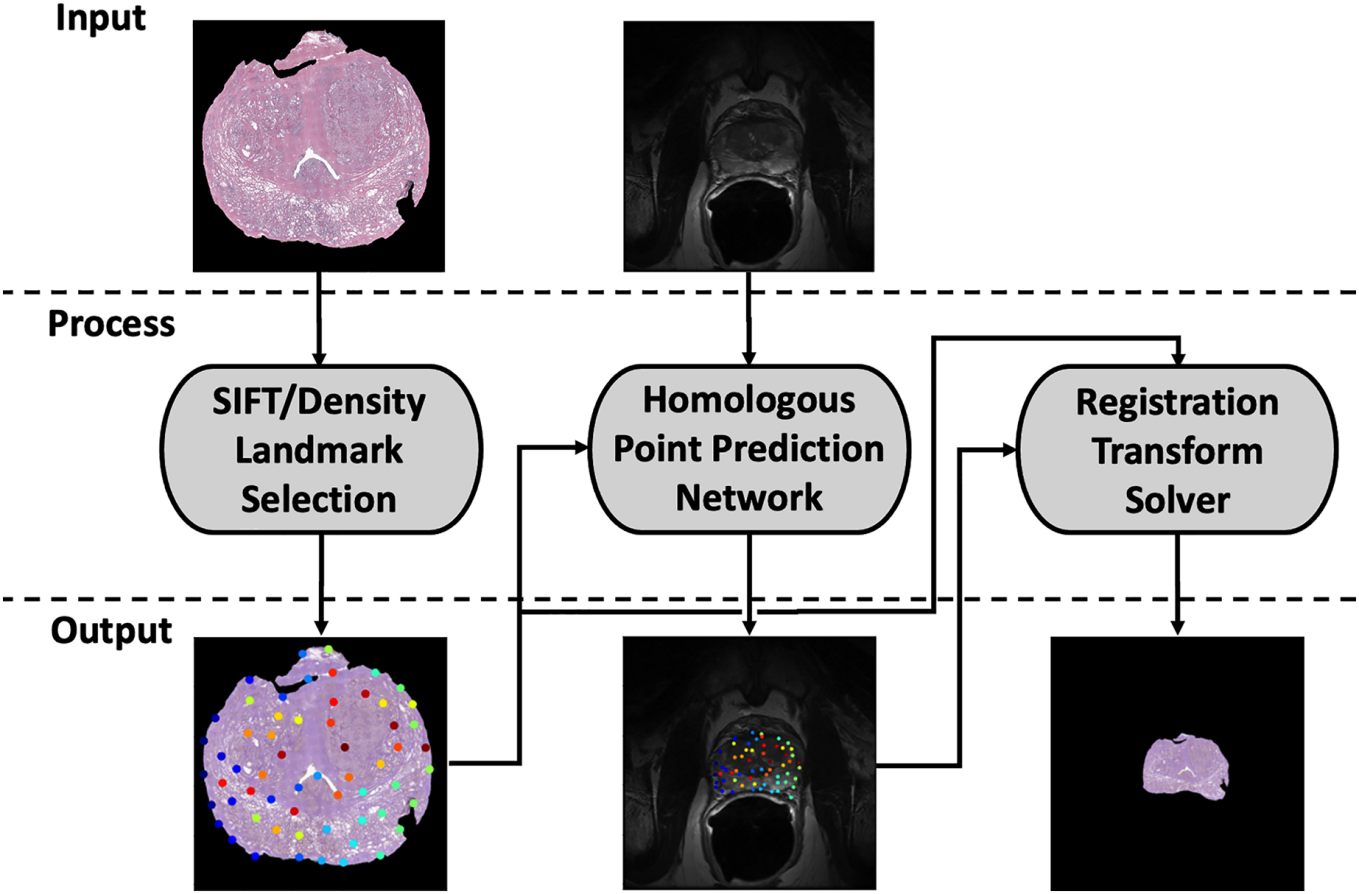
Demonstration of the image processing pipeline. Input to pipeline requires single slice histology image (moving) and corresponding 2-D MR slice (fixed) without masking. (Left) Solving for high information histology landmarks using a scale invariant feature transform (SIFT) algorithm and enforcing a specified point density. (Middle) Three head network design that accepts MR image, histology image, and proposed histology control points which then solves for corresponding MR homologous points. (Right) With homologous points solved in both domains, a homologous point registration algorithm (Thin-plate Spline) is applied and histology image is warped into MR space.

Our registration pipeline consists of three major components: a histology landmark generation algorithm, a homologous point prediction network, and a transformation resolver (Fig. 1). The landmark generation algorithm selects a set of points on the input histology image using scale invariant feature transform (SIFT) (Lowe, 2004) and enforcing a tunable point density. The homologous point prediction network uses three input heads that accept the original histology image (512 × 512), corresponding T2-weighted MR image (512 × 512), and the generated landmarks from step 1 to solve for the homologous landmarks in MR space. Finally, the set of histology-space input landmarks generated by the landmark generation algorithm and the paired set of MR-space homologous points predicted by the deep learning model are used by the transformation resolver to calculate a nonlinear transform (thin plate spline) that will warp the histology image to align with the MR image. This thin plate spline transform is finally applied to the histology image. The same transform may be applied to annotation maps derived in histology space to transfer pathological labels into MR space. All code and data used for this registration pipeline may be found at https://github.com/9crusher/homologous_point_prediction.

### Data collection

The data set for this project was obtained from the Medical College of Wisconsin. All patients were recruited prospectively with institutional review board approval (Medical College of Wisconsin Internal Review Board—PRO00022426) and written consent. Patients were scheduled for radical prostatectomy with clinical MRI scans acquired approximately 2 weeks prior to surgery.

We follow the procedure described by McGarry et al. (2018); following prostatectomy, the prostate tissue was formalin fixed, and a custom slicing jig derived from the MRI scans was created to obtain histology/MR slice correspondence. Whole mount prostate slides were stained with H&E (hematoxylin and eosin) and imaged on a microscope slide scanner (captured using 40× objective at 0.58 $\rm \mu$m/px). Manually-curated homologous control points were placed for all slides in the selected data set to act as ground truth. While full field MR images were used as input and training data for our model, prostate masks were also generated for use in calculating Dice coefficient (Dice, 1945; Sorenson, 1948) post training.

A total of 232 individual prostate slides from 45 patients were included in the study. Two patients were reserved for validation and seven were held-out for testing. The remaining patients comprised the training data set. Several slides included in the data set exhibit tears and/or folds. These artifacts were introduced during the slicing, staining, and/or mounting process.

### Landmark selection algorithm

The selection algorithm was used to generate a set of points (landmarks) with uniform coverage of the input histology images. To improve the chances of a network finding the same points in both the MR and histology image, the algorithm was designed to select “high information” points on the histology image. These points lie along the edges of the prostate, along the boundaries of the urethra and seminal vesicles, and along the edges of other significant structural features. The landmark selection algorithm is capable of producing point sets of varying density based on a configuration parameter.

The algorithm first selects candidate output points using the same difference of Gaussian-based method described in the original SIFT paper by Lowe (2004). Candidate points with contrast scores below an empirically-derived threshold of 0.04 are eliminated. This threshold was chosen because it led to sets of points that closely matched the point density seen in the manually-created point sets. Next, an edge score is calculated for each remaining candidate point. Edge scores are calculated as the ratio of the larger to the smaller eigenvalue of the Hessian matrix at the location of the candidate point in scale space. Rather than directly calculating these values, the algorithm uses the computationally-efficient method described in Harris & Stephens (1988) to estimate edge scores. Candidate points in the empirically-chosen top 19th percentile of edge score are classified as edges, and the remaining points are classified as interior feature points. Finally, a minimum distance between points is enforced through the sequential elimination of points within some user-defined distance of other points. Edge points are prioritized in this elimination process so interior points are deleted in the case of an edge and interior point minimum distance conflict. The output of this algorithm is a list of landmark points with uniform density and and complete coverage of the histological surface.

### Homologous point network

#### Preprocessing

Two images are required for network inference and training: the histology image (moving) and the corresponding T2-weighted MR image (fixed). The two input images were formatted as single-layer float tensors of shape 512 × 512. All intensity values in the images are scaled to a range of [0.0, 1.0] with the histology image converted from RGB to grayscale before the scaling procedure. Additionally, the fixed and moving images were zoomed so that the prostate was centered in the image with 50 pixels of padding on either side. The input point coordinates were adjusted to maintain their original positions on the zoomed image.

#### Network input

**Figure 2:**
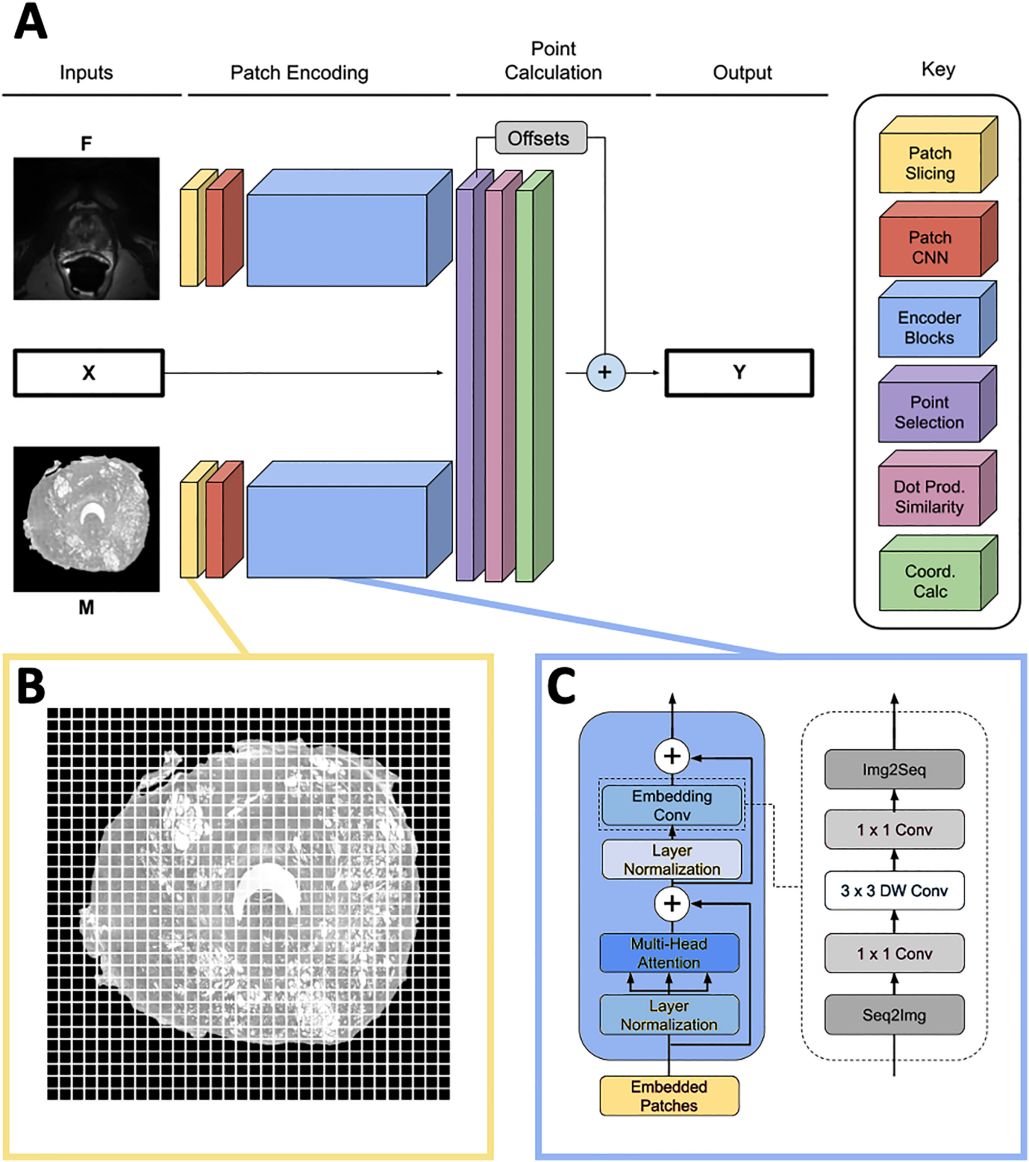
Network overview. (A) Full homologous point prediction network. The network has three input heads; M—Grayscale whole mount histology slide (512 × 512), F—Grayscale T2—weighted MR image (512 × 512), X—Histology landmarks. The output of the network is the set of homologous points in MR space—Y. (B) Histology and MR images are divided into patches, prior to linear embedding (MR image shown). The number of patches is a function of the number of points found in the histology landmark generation step. The transformer then uses the linear patch embeddings to compare histology and MR images to find similar patches and propose homologous points. (C) Locality-preserving transformer encoder block that uses convolution as opposed to the commonly-used global multi-head mechanism.

The network predicts the homologous points in MR space for some set of input landmark coordinates ${\bf X} \in {{\mathbb R}^{L \times 2}}$ where *L* is the number of points. It contains three input heads: one for the input point coordinates **X**, one for the moving image ${\bf M} \in {{\mathbb R}^{512 \times 512}}$ and one for the fixed image ${\bf F} \in {{\mathbb R}^{512 \times 512}}$. The output is a set of homologous point coordinates ${\bf Y} \in {{\mathbb R}^{L \times 2}}$ in MR space (Fig. 2A). Because *L* must be fixed for each input in a batch and point sets generated by the SIFT algorithm may have varying numbers of points, *L* is set to a number larger than the maximum expected number of points, and **X** is padded with [0, 0] points to achieve length *L* for every input. In practice, we found *L* = 75 to be large enough to contain all points generated by the point placement algorithm.

#### Patch encoding

Input images **M** and **F** are sliced into size p × p patches as seen in Fig. 2B, and each patch is passed into a patch CNN consisting of three 2D convolutional layers. The first two layers contain 64 kernels of shape 2 × 2 and stride 2 × 2, and the final layer is of shape $\textstyle{p \over 4}$ with d kernels. This serves to convert each patch in the input space to a feature vector of length d. A learned position embedding is added to this vector. The result of applying this operation to the moving and fixed image patches is two tensors ${{\bf P}_m}$, ${{\bf P}_f} \in {{\mathbb R}^{N \times d}}$ containing embeddings for **M** and **F** respectively where $N = {\left( {\displaystyle{{512} \over p}} \right)^2}$, the number of patches per image.

The patch encoder blocks transform the vector representations of the image patches ${{\bf P}_f}$, ${{\bf P}_m} \in {{\mathbb R}^{N \times d}}$ into embeddings ${\widehat {\bf P}_f}$, ${\widehat {\bf P}_m}$ representing location on the prostate structure. We use the locality-preserving encoder block (Fig. 2C) first proposed by Li et al. (2021) (LocalViT). This block is a modified version of the global encoder blocks used in Dosovitskiy et al. (2020) (ViT). Locality-preserving encoder blocks use the same global multihead attention mechanism as traditional transformer blocks. However, the traditional block’s subsequent MLP layers are replaced with convolution. The locality-preserving portion of the block reshapes the embeddings from ${{\mathbb R}^{N \times d}}$ to ${{\mathbb R}^{\sqrt N \times \sqrt N \times d}}$ so that the two dimensional relative positioning of the input image patches is mirrored in the relative positioning of the embeddings. Two dimensional convolution with a 1 × 1 kernel is run on the reshaped embeddings resulting in a ${{\mathbb R}^{\sqrt N \times \sqrt N \times 4d}}$ tensor. Next, 3 × 3 depthwise convolution is performed on the expanded embeddings before the result is squeezed to ${{\mathbb R}^{\sqrt N \times \sqrt N \times d}}$ with 1 × 1 convolution. Finally, the tensor is reshaped to ${{\mathbb R}^{N \times d}}$.

Our model uses two transformer encoders composed of the blocks described above. One of the encoders encodes histology patches and the other encodes MRI patches; weights are not shared between the two branches. Each transformer encoder consists of six local blocks using d = 128.

#### Point calculation blocks

After encoding, the histology output is further refined for point selection. ${\widehat {\bf P}_m}$ is reduced (1) to a new list of encodings ${\bf P^\prime}_m \in {{\mathbb R}^{L \times d}}$ where only the encodings corresponding to the patches of **M** overlapping with landmarks from **X** are kept.

(1) $${{\bf P}^\prime }_m = \bigg \{ {\mkern 1mu} {\widehat {\bf P}_m}\bigg [floor\bigg (\displaystyle{{{\bf X}[i]} \over p}\bigg)\bigg]{\rm \mid }i = 0,{\rm }...,{\rm }L - 1\bigg\}$$

The offsets of the landmarks from the centers of their corresponding patches (2) are also calculated at this stage of the model.

(2) $${\bf O} = ({\bf X}\quad {\rm mod}\; p) - floor\bigg(\displaystyle{p \over 2}\bigg)$$

Next, the dot product similarity **S** $\in {{\mathbb R}^{L \times N}}$ between the landmark patch embeddings in ${\bf P^\prime}_m$ and all of the patch embeddings in ${\widehat {\bf P}_f}$ is calculated. Each value ${{\bf S}_{ij}}$ in ${\bf S}$ may be considered a representation of the similarity between the patch of **M** corresponding to landmark ${\bf X}[i]$ and patch *j* from **F**.

(3) $${\bf S} = Softmax({\bf P^\prime}_m {({{\bf P}_f})^T})$$

Each row of **S** is multiplied by a meshgrid of y and x coordinates constructed with range $\left[ {\displaystyle{p \over 2},512 - \displaystyle{p \over 2}} \right]$ with spacing of p. This yields weighted averages of coordinates $\hat {\bf Y} \in {{\mathbb R}^{{\bf L} \times {\bf 2}}}$ centered at the area of highest similarity between each embedded patch in ${\bf P^\prime}_m$ and the patches of **F**. It may be helpful to consider the calculation of $\hat {\bf Y}$ as the result of dot product attention in which the queries are embedded patches from **M**, the keys are embedded patches from **F**, and the values are the coordinate of the patch centers.

Finally, output **Y** is calculated by adding **O** to $\widehat {\bf Y}$. This output is masked so that all padding landmarks in **X**—the points set to [0, 0] in the input—are set to [0, 0] in the output. An overview of the complete network structure may be seen in Fig. 2.

### Model training

The homologous point prediction model was trained using the ground truths of manually-placed homologous points in MR and histology space. Mean absolute error between the predicted points and the ground truth points was used as the loss function. The network was trained for 128 epochs with the data augmentation described below. The model contains 5,371,904 trainable parameters and was run on a NVIDIA Tesla T4 GPU using the Tensorflow and Keras Python frameworks.

#### Data augmentation

To prevent overfitting to the 36-patient training set, image augmentation was used. Random thin plate spline (TPS) augmentations with a maximum point deviation of 16 pixels were applied to both the fixed and moving images, and the ground truth points were warped through these transforms to maintain the same positions on the prostates. Random rotations in the range of [−20, 20] degrees were applied to the fixed images.

#### Mono-modal and multi-modal training schema

Fifty percent of the fixed/moving image pairs passed to the model each epoch were from the same modality. In the same-modality case, either a histology or MR slide was selected; the human-placed points on that slide were used as both the input landmarks and the correct outputs for the training example. Because of the augmentations described above, the points and images would be different in the fixed and moving branches of the model, so the training example was useful for generalization. Furthermore, the patch encoding (Fig. 2) branches of the network were actively switched depending on the modalities of the images passed into each branch. Both an MR and a histology patch branch were maintained during the training. When a same-modality histology training example was passed into the network, the histology patch encoding branch was used as both the fixed and moving branch. The same was true for the MR patch encoding branch for a same-modality MR training example. The default case of the network uses the MR encoding branch in as the fixed branch and the histology encoding branch as the moving branch.

#### Evaluation metrics

Average control point deviation and Dice coefficient were used to quantitatively evaluate the registration performance. To calculate average control point deviation, a registration pipeline was used to solve for a transform from histology space to MR space. The human-placed landmarks in histology space were transformed to MR space using the solved transform. Finally, the average Euclidean distance between every pair of warped landmarks and ground truth MR points was calculated. Because the ground truth control points were placed on both the edges and interior of the prostate structure, this metric quantifies the edge and internal structural alignment of a registration result.

For clarification, when evaluating the homologous point pipeline with the control point deviation metric, two distinct point sets were at play. (1) The set of points placed by the SIFT algorithm and predicted in MR space by the deep learning network and (2) the human annotated landmarks in histology space and their corresponding human annotated landmarks in MR space. The quality of the resolved transform was evaluated using the human-placed ground truths.

**Figure 3:**
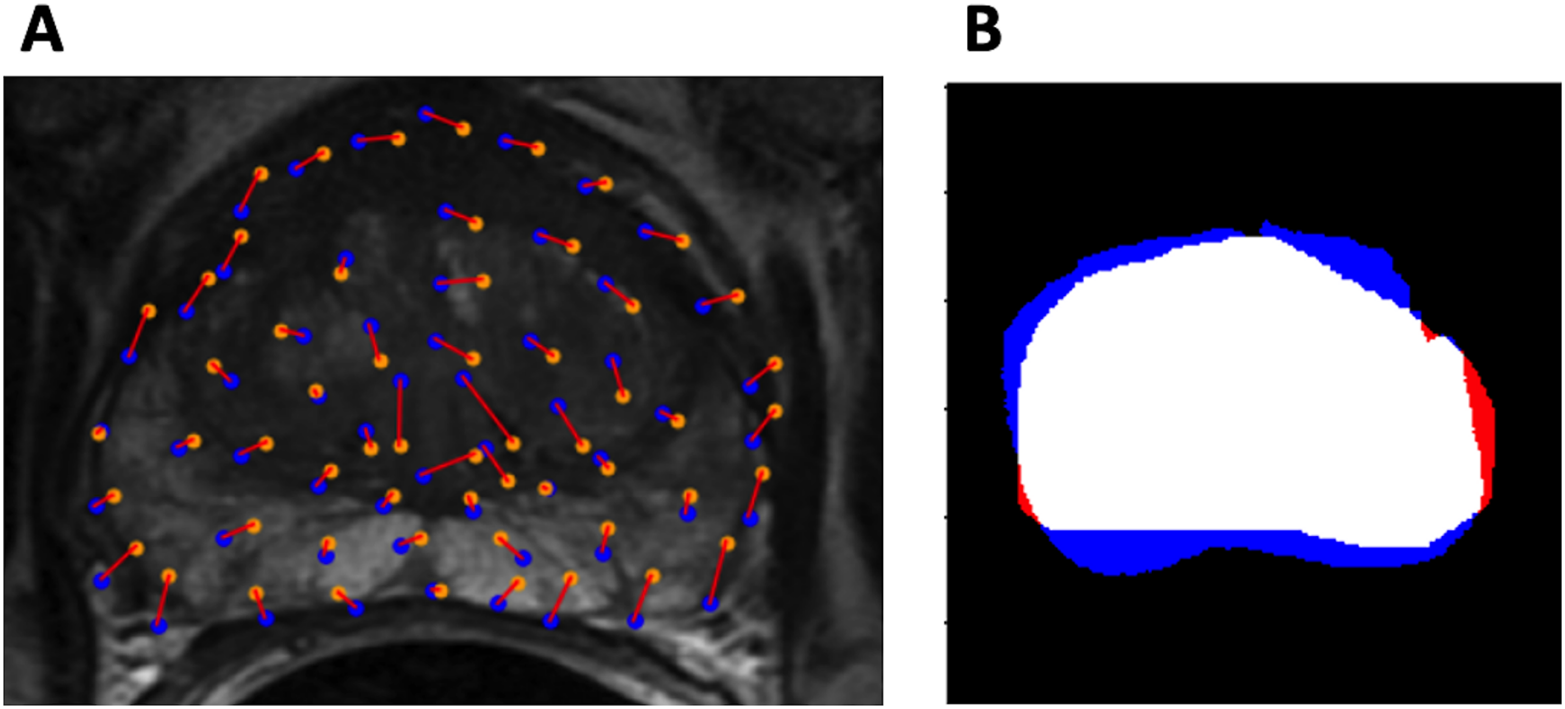
Evaluation metrics. (A) Example control point deviation. Average control point deviation is the mean length of the red lines between the ground truth points (blue) and the warped landmarks (orange). (B) Example Dice coefficient. *M_A_* is red, *M_B_* is blue, *M_A_* $\cap$*M_B_* is white.

Dice coefficient was also used to evaluate model performance. Visualizations of the evaluation metrics are shown in Fig. 3.

#### Statistical tests

Training, validation, and testing images were held constant for all comparisons made. Repeated Measures ANOVA was used to compare evaluation metrics between all models. Mauchly’s test of sphericity was used to detect violations of the sphericity assumption. Where sphericity violations occured, Huynh-Feldt sphericity correction was applied. *Post-hoc* tests were performed with Bonferroni correction. An $\alpha$ level of 0.05 was used to test for significance. All statistical tests were performed in JASP 0.16.2.

## Results

### Softmax output—point placement visualization

**Figure 4:**
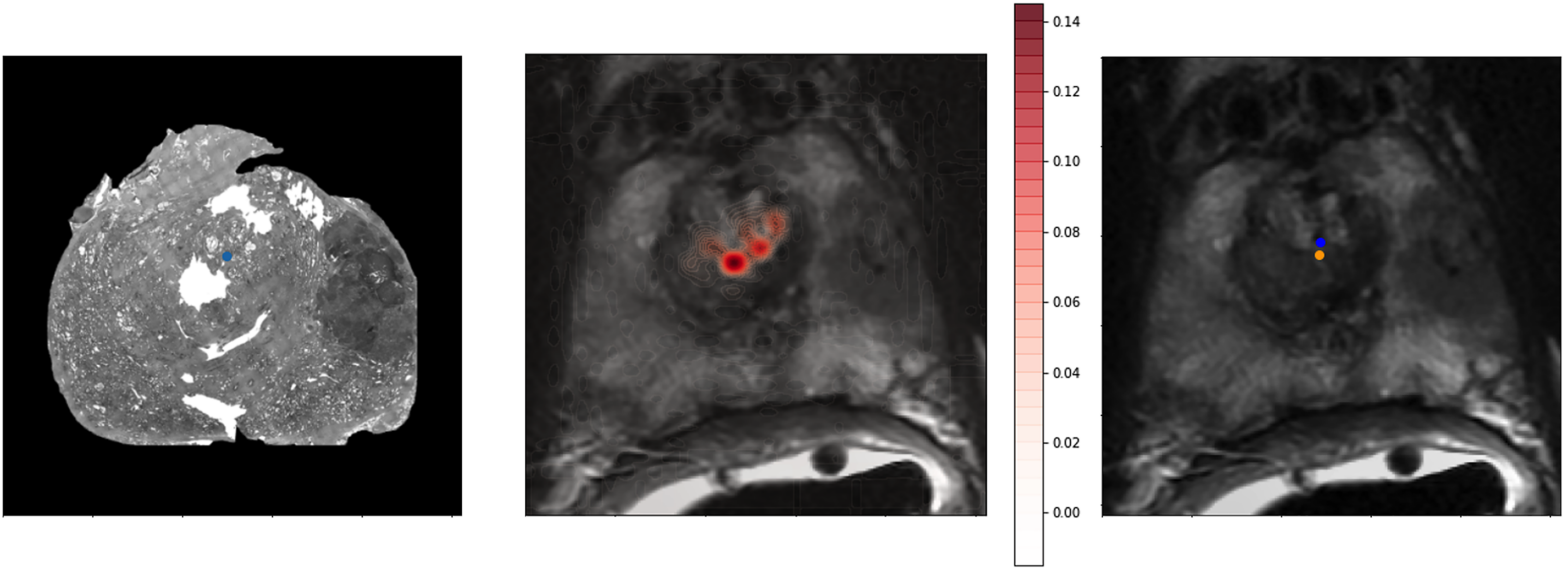
Softmax output visualization for a single point. Left—Original histology with single landmark (blue). Center—Corresponding MR slice with predicted point probability heatmap generated from the network. The predicted point probability is used to generate the estimated location of the originally placed histology landmark in MR space. Right—Corresponding MR slice with blue predicted point *vs*. yellow ground truth point.

To provide a better qualitative interpretation of how the network resolves the homologous points, we visualized the softmax layer output for a single homologous point in histology space—Fig. 4 (left). Figure 4 (center) depicts a single row of **S** projected back into the two dimensional space of **F**. To calculate a row in **Y**, the network resolves the centroid of all patches in the image modified by the depicted weights. This centroid is then further shifted by the original offset of the landmark in the histology side patch. This results in the predicted location of the homologous point in the fixed image—Fig. 4.

### Method comparison

The proposed homologous point pipeline was compared to thin plate spline transforms resolved with human ground truth points and to several versions of the ProsRegNet, the current state-of-the-art model. The highest-performing published weights for ProsRegNet were used in the evaluation (ProsRegNet—Paper). Additionally, ProsRegNet was configured to train on the same supercomputer as the homologous point pipeline and was finetuned with a hyperparameter grid search on the same training data set. The highest performing finetuned ProsRegNet model (ProsRegNet—Tuned) was selected for this comparison. This step was performed to maximize the performance of ProsRegNet and allow it to generalize on the same cohort as the homologous point pipeline. All four models were evaluated for both Dice coefficient and mean control point deviation on the same held-out test set.

**Figure 5:**
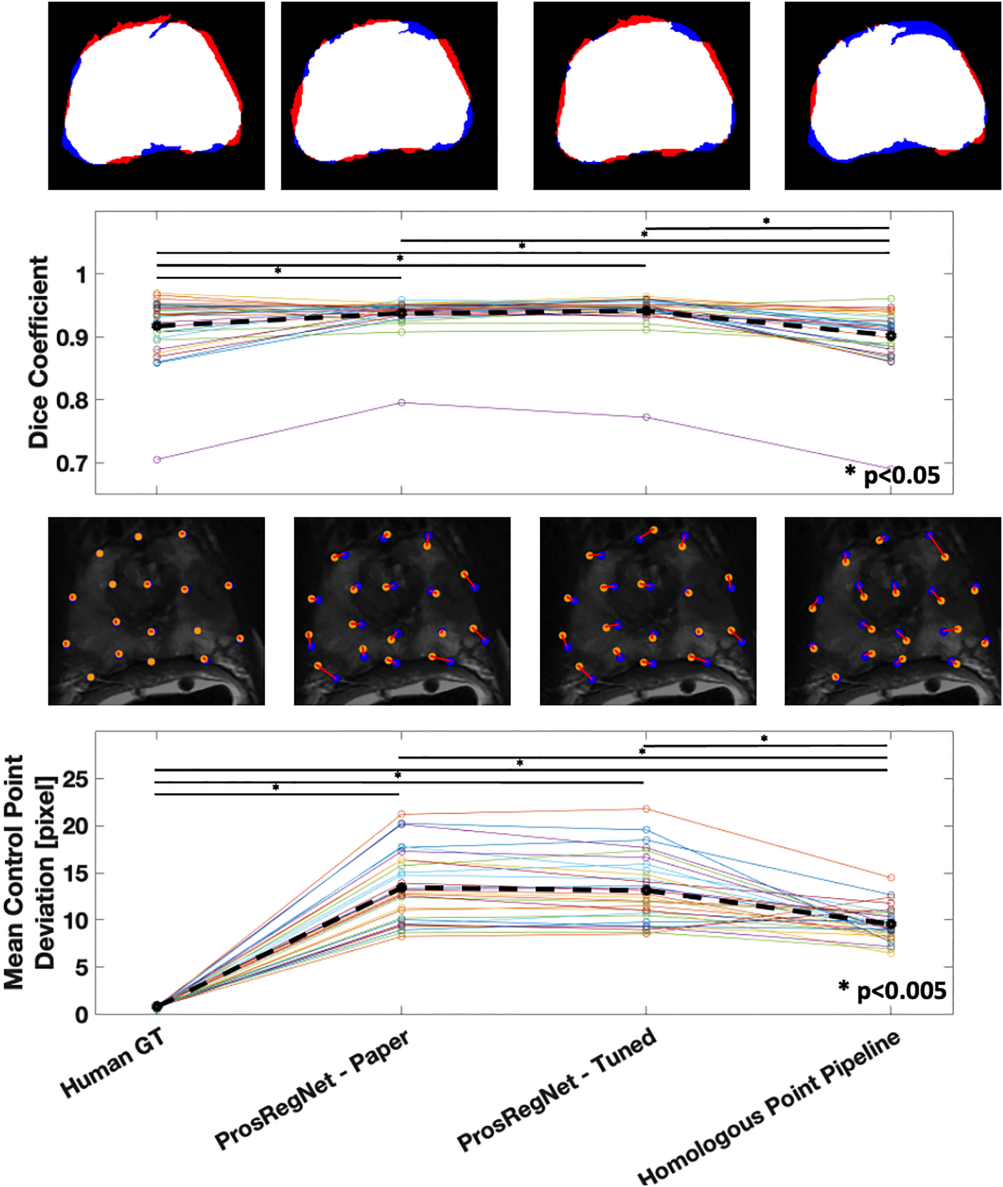
Comparison of registration results. Method evaluations from left to right; Human Ground Truth, ProsRegNet—Paper, ProsRegNet—Tuned, and Homologous Point Pipeline. Top— visualization of the Dice coefficient. Blue indicates the ground truth prostate mask in MR space, red indicates prostate mask of histology post registration, and white denotes overlap between the two masks. Top middle—Dice coefficients with individual prostate slides shown in color, black dotted line represents average Dice coefficient for each method. Bottom middle—visualization of the control point displacement between transformed histology space ground truth and MR space ground truth. Yellow dots mark MR point ground truth, blue dots mark transformed histology landmarks, red lines indicate error. Bottom—Mean control point deviation with individual prostate slides shown in color, black dotted line represents average control point deviation for each method. *p*-value < 0.005 marked with ‘*’.

The average Dice coefficient for the homologous point pipeline was found to be $0.90 \pm 0.05$, which under-performed Prosregnet—Paper at $0.94 \pm 0.03$ $(p < 0.001)$, Prosregnet—Tuned at $0.94 \pm 0.03$ $(p < 0.001)$, and the Human GT at $0.92 \pm 0.05$ $(p = 0.012)$ (Fig. 5—Top).

Mean control point deviation in the homologous point pipeline was found to be $9.55 \pm 1.76$ pixels which outperformed both ProsRegNet—Paper at $13.44 \pm 3.71$ pixels $(p < 0.001)$ and ProsRegNet—Tuned at $13.12 \pm 3.49$ pixels $(p < 0.001)$ but fell short of the Human GT at $0.81 \pm 0.16$ pixels $(p < 0.001)$ (Fig. 5—Bottom).

It should be noted that the ProsRegNet pipeline requires masked MR images to resolve transforms while the homologous point pipeline registered unmasked images. Additionally, the control point deviation of the human ground truth registrations was near zero because this registration method directly used the ground truths to resolve TPS transforms which due to degrees of freedom may not perfectly fit all control points.

## Discussion

We propose a new automatic prostate image registration pipeline that uses a transformer-based deep learning model to predict homologous points on image pairs and resolves a transform based on the predicted points. The homologous point pipeline is transform invariant; any transform type that can be resolved from two point sets may be used for the registration. Additionally, our pipeline is able to resolve transforms without any masking.

The core of our homologous point pipeline is a network that predicts a set of points that correspond to a set of landmarks selected on a histology image. The network was trained to minimize control point deviation—a metric that quantifies both internal and edge alignment of a registration result. This is in contrast to ProsRegNet which optimizes directly for Dice coefficient. The homologous point pipeline scores better than ProsRegNet in average control point deviation while performing slightly worse in Dice coefficient. Additionally, we note that ProsRegNet has been previously shown to outperform existing registration pipelines such as RAPSODI. In future studies, it may make sense to incorporate both control point deviation and Dice coefficient into the objective function. However, this would require organs to be easily delineated in both imaging domains and could prohibit registration of partial tissue specimens to whole organs.

Due to the limited size of the data set used to train the point prediction network, a novel approach was taken to expand the effective size of the training set while properly training the modality-coupled branches of the network. One half of the training examples passed into the network were differently-warped versions of the same image—either histology or MR. The patch CNNs used in both branches of the network were actively toggled so that the weights learned for one modality were never used for the other. For example, in the case of a training example with two MR images, the same patch CNN was used for both branches. In the base use case of the network, the MR patch CNN was used for the moving image and the histology patch CNN was used for the fixed image. This training scheme allowed for a greatly increased number of training examples while preserving the modality coupling of the network branches.

Data limitations also prevented the evaluation our model’s resilience to torn, folded, and partial histological specimens. We posit that the unique approach of identifying correspondence to only specific landmarks on histology images allows our pipeline to align partial slides with just a portion of a prostate in MR space. The prevalence of damaged histology slides in clinical data makes this an important trait for an automatic registration method. Existing automated registration techniques attempt to register partial prostate slides to overlap with the entire prostate in MR space. Unless accounted for, this could lead to overestimation of Dice coefficients. The true control point correspondence of aligning the slide with only part of the prostate in the MR image could lead to a better characterization of the model’s ability to register images. It is proposed that this trait makes the homologous point pipeline highly portable to medical image registration tasks where only partial tissue samples are available. However, future experiments are needed to evaluate this application.

### Future directions

With our homologous point pipeline established, we envision three broad categories of future work. First, the pipeline should be characterized on frequently seen histological artifacts; we have anecdotally observed encouraging performance with partial tissue samples and tears in the current data set. However, the performance on slides with several different types of artifacts including partial tissue sections, tearing, and folding should be quantified. These artifacts are commonly introduced during tissue processing and may exclude a significant number of samples from analysis. Second, this approach should be fine-tuned and tested on additional organ systems. In this study, the homologous point pipeline was only characterized for the prostate data, but it is designed to adapt easily to other data. Adaption of this pipeline to the brain is especially enticing due the size of the organ often limiting histological processing to partial tissue sections. To the best of our knowledge, other current approaches can not be adapted to resolve points between partial tissue samples and whole organ imaging. Finally, this current approach requires the given histology and MR slices to be paired at the input. Both the histology and MR samples may include three-dimensional relationships that this approach may not currently leverage. If this approach were adapted to organ volumes/voxels, we may improve homologous point predictions and eliminate the required pre-processing of tissue to MR slice pairing.

## Conclusions

We proposed a new automatic prostate image registration pipeline that predicts homologous points to resolve a nonlinear transform. The method was designed to maximize internal and edge prostate landmark correspondence in its registration results. Our pipeline achieved better average control point deviation with slightly worse Dice coefficient on unmasked data than the current state of the art achieved using masked data. The improved control point deviation is critical in a clinical research setting because control point deviation directly impacts the accuracy of the cancer annotations mapped by the registration. The ability to operate on unmasked data reduces the amount of manual intervention required to register a set of images; further, without this restriction the network may be more robust to partial or torn samples.

## Additional Information and Declarations

### Data Availability

The following information was supplied regarding data availability: The model is available at GitHub: https://github.com/9crusher/homologous_point_prediction; 9crusher. (2022). 9crusher/homologous_point_prediction: V1.0.1 (v1.0.1). Zenodo. https://doi.org/10.5281/zenodo.7302497. The data used for model is available at figshare: Ruchti, Alexander; Neuwirth, Alexander; Lowman, Allison; Duenweg, Savannah; LaViolette, Peter; Bukowy, John (2022): Data supporting Homologous Point Transformer for Multi-modality Prostate Image Registration. figshare. Dataset. https://doi.org/10.6084/m9.figshare.20756581.v1.
